# Visiting Hour Policies in Intensive Care Units, Southern Iran

**DOI:** 10.5812/kowsar.20741804.2242

**Published:** 2011-09-15

**Authors:** S Haghbin, Z Tayebi, A Abbasian, H Haghbin

**Affiliations:** 1 Department of Pediatrics, Pediatric Intensive Care Unit, Nemazee Hospital, Shiraz University of Medical Sciences, Shiraz, Iran; 2 Department of Nursing and Midwifery, Tehran University of Medical Sciences, Iran; 3 Department of Statistics, Shiraz University, Shiraz, Iran

**Keywords:** Visiting policy, Intensive care unit, Visiting hours, Iran

Dear Editor,

Admission to intensive care unit (ICU) is potentially a stressful experience for both the patients and their families. In addition to pain and severity of the critical diseases, sleeplessness, immobility and overwhelming noises from ICU equipments, anxiety from overhearing the stranger and unfamiliar conversations of the staff and visitors about the patients’ conditions, psychologically affect the ICU patient.[1][2][3] The critical condition of the patients could be a source of stress for the respective families as well, so that sometimes it is referred to as in-family crisis.[4][5] Nowadays, the role of families in the critical patients’ recovery is considered very important, and professionals focus on family and patient-centered systems. Therefore, it has long been suggested that open and flexible visiting policies can positively affect such patients’ conditions and consequently can help families cope with the crisis and promote their satisfaction.

To investigate the current status of the visiting hours and policies, we carried out this descriptive cross-sectional study in summer 2009 in all 71 ICUs in Fars Province, south of Iran. A questionnaire based on a previous study, conducted in Italy,[3] was prepared and filled for each unit by a single researcher through face to face and telephone communication with respective head nurses. To this end, the ICUs consisting of general (20%), specialist (17%), neonatal/pediatric (18%) and cardiac (38%) units in private centers, university teaching and governmental non-teaching hospitals were enrolled. As the data were shown in the Table 1, restrictions were on the numbers and age of visitors, and hours of visits in all units. In 39.4% of the units no visits was allowed. In 15.5%, visiting time was 1.5-2 hours and in 23.9% was one hour a day in some of which the visits were through the glass windows due to the limitation of space and facilities.

**Table 1:** Visiting policies in the surveyed intensive care units, Fars, southern Iran, 2009

**Open policy**		**Number**	**%**
	Yes	7	9.9
	No	64	90.1
Daily visiting time			
	No visitation	28	39.4
	Up to 1 hour	17	23.9
	1.5-2 hour	11	15.5
	>2 hours	15	21.2
Frequency of visit per week			
	0 days	28	39.4
	2 days	2	2.8
	3 days	1	1.4
	Every day	40	56.4
Number of visitors at a time			
	0 person	28	39.4
	One person	21	29.6
	2 persons	22	31
Visit by children permitted			
	Yes	0	0
	No	66	93
	Sometimes	5	7
In-charge of visiting hours			
	Nurse in-charge	16	22.5
	Physician	7	9.9
	Hospital authorities	40	56.3
	Group decision	3	4.2
	Unknown	5	7.1
Decision on exceptional events			
	Nurse in-charge	48	67.6
	Physician	10	14.1
	both	13	18.3
Telephone Information provision			
	Yes	65	91.6
	No	6	8.4
The person receiving telephone information			
	No one	6	8.4
	Immediate family	19	26.8
	Relatives	46	64.8

The main finding of the present survey was the uniform practicing restriction on the visiting policy in all ICUs. Gianini et al. similarly conducted a study in Italy and found almost the same results.[6] Tendency toward open policy has been reported from France (23%), UK (50%), and Sweden (70%).[7] These varying visiting policies might be due to different cultural and attitudinal factors in different communities.

Restricted visiting hours dates back to the late 1800s and for a variety of reasons including creating more discipline in the wards and avoiding the transmission of infection.[8][9] Berti et al. revealed that the influential environmental and organizational factors in this respect include the limitation of ICU space, providing more time to patients’ rest,[10] insufficient nurse to bed ratio, avoiding interruption of nursing care and reducing the tension between the staff and families. Also, researchers in the field unanimously agree that the attitudes of the ICU staff is the most important factor that can facilitate the path towards unrestricted visiting policy and a commitment to removing all barriers.[3]

Considering the evidence supporting open policies and the present study findings, we can conclude that the revision of current visiting policies in Iran is required in order to create more positive effects and satisfaction on the part of patients and their families. We do not recommend the universal implementation of unrestricted ICU visiting policies but rather a kind of modification in the policies, based on our cultural background so that a balance is established between the patients’ safety, their families’ need and also the nurses and physicians’ management in the ICUs. To do so, further studies need to be carried out to identify the obstacles to the implementation of open visiting policies.
